# Tat-GSTpi Inhibits Dopaminergic Cells against MPP^+^-Induced Cellular Damage via the Reduction of Oxidative Stress and MAPK Activation

**DOI:** 10.3390/biomedicines11030836

**Published:** 2023-03-09

**Authors:** Yeon Joo Choi, Hyeon Ji Yeo, Min Jea Shin, Gi Soo Youn, Jung Hwan Park, Eun Ji Yeo, Hyun Jung Kwon, Lee Re Lee, Na Yeon Kim, Su Yeon Kwon, Su Min Kim, Dae Won Kim, Hyo Young Jung, Oh-Shin Kwon, Chan Hee Lee, Jong Kook Park, Keun Wook Lee, Kyu Hyung Han, Jinseu Park, Won Sik Eum, Soo Young Choi

**Affiliations:** 1 Department of Biomedical Science, Research Institute of Bioscience and Biotechnology, Hallym University, Chuncheon 24252, Republic of Korea; 2 Department of Biochemistry and Molecular Biology, Research Institute of Oral Sciences, College of Dentistry, Gangneung-Wonju National University, Gangneung 25457, Republic of Korea; 3 Department of Veterinary Medicine & Institute of Veterinary Science, Chungnam National University, Daejeon 34134, Republic of Korea; 4 School of Life Sciences, College of Natural Sciences, Kyungpook National University, Taegu 41566, Republic of Korea

**Keywords:** Tat-GSTpi, PD, neuroprotection, MAPK, protein therapy

## Abstract

Glutathione S-transferase pi (GSTpi) is a member of the GST family and plays many critical roles in cellular processes, including anti-oxidative and signal transduction. However, the role of anti-oxidant enzyme GSTpi against dopaminergic neuronal cell death has not been fully investigated. In the present study, we investigated the roles of cell permeable Tat-GSTpi fusion protein in a SH-SY5Y cell and a Parkinson’s disease (PD) mouse model. In the 1-methyl-4-phenylpyridinium (MPP^+^)-exposed cells, Tat-GSTpi protein decreased DNA damage and reactive oxygen species (ROS) generation. Furthermore, this fusion protein increased cell viability by regulating MAPKs, Bcl-2, and Bax signaling. In addition, Tat-GSTpi protein delivered into the substantia nigra (SN) of mice brains protected dopaminergic neuronal cell death in the 1-methyl-4-phenyl-1,2,3,6-tetrahydropyridine (MPTP)-induced PD animal model. Our results indicate that the Tat-GSTpi protein inhibited cell death from MPP^+^- and MPTP-induced damage, suggesting that it plays a protective role during the loss of dopaminergic neurons in PD and that it could help to identify the mechanism responsible for neurodegenerative diseases, including PD.

## 1. Introduction

Parkinson’s disease (PD) is a common neurodegenerative disease. The main feature of PD is the loss of midbrain dopaminergic neurons in the substantia nigra (SN). It is well known that aging and oxidative stress are major pathogenetic risk factors for PD, which affects about 0.3% of the population in developed countries [1,2,3]. Previous studies have shown that mice are highly susceptible to the neurotoxic effects of the toxicant 1-methyl-4-phenyl-1,2,3,6-tetrahydropyridine (MPTP), which selectively depletes dopamine and has been used to generate models of PD [4,5,6].

Excessive generation of oxidative stress-induced reactive oxygen species (ROS) contributes to neuronal diseases such as PD [7,8]. Moreover, the oxidative stress-induced ROS activation and mitogen-activated protein kinase (MAPK) signaling pathways are involved in MPTP-induced PD animal models and human PD patients. This suggests that the regulation of MAPK signaling pathways is a key strategy for protecting against dopaminergic neuronal cell death induced by oxidative stress [9,10].

Glutathione S-transferase (GST) exists in three forms: the basic hepatic form (alpha), the near-neutral hepatic form (mu), and the acidic placental form (pi). This protein is abundant in various tissues, such as kidneys and human liver cancer tissues [11,12,13,14,15]. Several reports have revealed that GSTpi proteins play a crucial role in cell survival against carcinogens and cytotoxins. GSTpi proteins also regulates cellular signaling pathways, such as c-Jun NH2-terminal kinases (JNKs), and inhibits tumor necrosis factor-alpha (TNFα)-induced apoptosis [16,17,18,19]. Furthermore, other reports have shown that GSTpi protein levels are significantly increased in SN or leukocytes of PD patients and suggested that GSTpi may be biomarker for PD [20,21]. In contrast, reduction of GSTpi protein levels in MPTP-induced animal model increases sensitivity to MPTP, suggesting that the expression of GSTpi proteins may influence the pathogenesis of ROS-induced neuronal disorder, including PD [22]. Furthermore, the inhibition of GSTpi protein expression leads to increased oxidative stress, JNK activation, and, finally, degeneration of the dopaminergic neurons [23]. However, the exact function of the GSTpi protein in PD has not yet been revealed.

The protein transduction domain (PTD), including Tat, can deliver proteins to cells and the brain tissue. Previous reports have shown that various cell permeable Tat fusion proteins have protective effects against oxidative stress-induced cell damage [24,25,26,27,28,29,30,31]. In the present study, we investigated the effects of Tat-GSTpi proteins on MPP^+^- and MPTP-induced SH-SY5Y cells and a PD animal model and observed whether this fusion protein could protect against oxidative stress-induced cell death. However, more studies on the effect of GSTpi proteins on PD are needed. 

## 2. Materials and Methods

### 2.1. Materials and Cell Culture

The antibodies used in this study are summarized in Table 1. Unless otherwise stated, all other reagents were of the highest grade available. Cell culture was performed as described previously in [31].

### 2.2. Delivery of Tat-GSTpi Protein to SH-SY5Y Cells

The delivery of Tat-GSTpi proteins to the cells and the subsequent detection of this protein were performed according to the previously described methods of [28,32,33].

### 2.3. Cell Viability Assay 

Cell viability was confirmed by MTT assay [28,34]. Briefly, Tat-GSTpi, GSTpi, and Tat peptide (0.5–3 μM) were treated for 1 h in SH-SY5Y cells and MPP+ (5 mM) was treated for 14 h. Then, the OD value was read by an ELISA microplate reader (Multiskan MCC/340; Thermo Labsystems Oy., Helsinki, Finland) at 450 nm.

### 2.4. Analysis of Intracellular ROS and TUNENL Staining

The levels of ROS production and DNA damage were determined by DCF-DA (Sigma-Aldrich, St. Louis, MO, USA) and TUNEL (Roche Applied Science, Basel, Switzerland) staining as described in [28,35].

### 2.5. Experimental Animals and Treatment

All experimental procedures were approved by Hallym University [Hallym 2020-30]. Male C57BL/6 mice received MPTP to prepare a PD model according to procedures reported in [36,37]. The mice were divided into five groups (*n* = 7/each group) and were intraperitoneally (i.p.) injected with Tat-GSTpi protein (2 mg/kg) 12 h before the MPTP treatment. Then, the mice were killed 1 week after the last injection.

### 2.6. Immunohistochemistry

Immunohistochemistry was carried out using methods detailed in [36,37]. Briefly, the brain sample sections (30 μm) were incubated with 3% bovine serum albumin in PBS for 30 min. Then, the sample sections were incubated with a His or tyrosine hydroxylase (TH) antibody for the detection of Tat-GSTpi proteins and DA neurons. To detect viable cells, cresyl violet counterstaining for Nissle bodies was conducted after TH immunostaining. The image of each section was analyzed by a blinded observer.

### 2.7. Statistical Analysis

Data are expressed as the mean ± SEM of three different experiments. Differences between groups were analyzed using a one-way analysis of variance (ANOVA) followed by a Bonferroni’s post hoc test. A value of *p* < 0.05 was considered as indicating a statistically significant difference.

## 3. Results

### 3.1. Tat-GSTpi Protein Delivered to SH-SY5Y Cells

**Figure 1:**
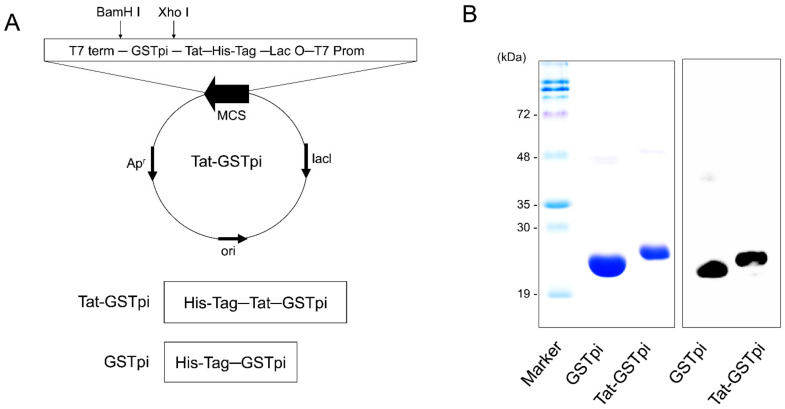
Construction and purification of Tat-GSTpi protein. (**A**) Schematic sequence of Tat-GSTpi and GSTpi protein. Tat-GSTpi protein consists of six histidine, Tat peptide, and cDNA of human GSTpi; (**B**) Purification of Tat-GSTpi and GSTpi proteins were analyzed by SDS-PAGE and Western blotting with an anti-histidine antibody.

As shown in Figure 1A, we constructed a PTD-GSTpi fusion protein which includes six consecutive His residues, as described in [32]. To obtain pure fusion proteins, we induced fusion protein expression with 0.5 mM IPTG and the proteins were purified by Ni^2+^-NTA and PD-10 columns (Qiagen; Valencia, CA, USA). The purified proteins appeared as a single band, and we confirmed this using a Western blot analysis with anti-His antibody, since the proteins included six consecutive His residues (Figure 1B,C).

**Figure 2:**
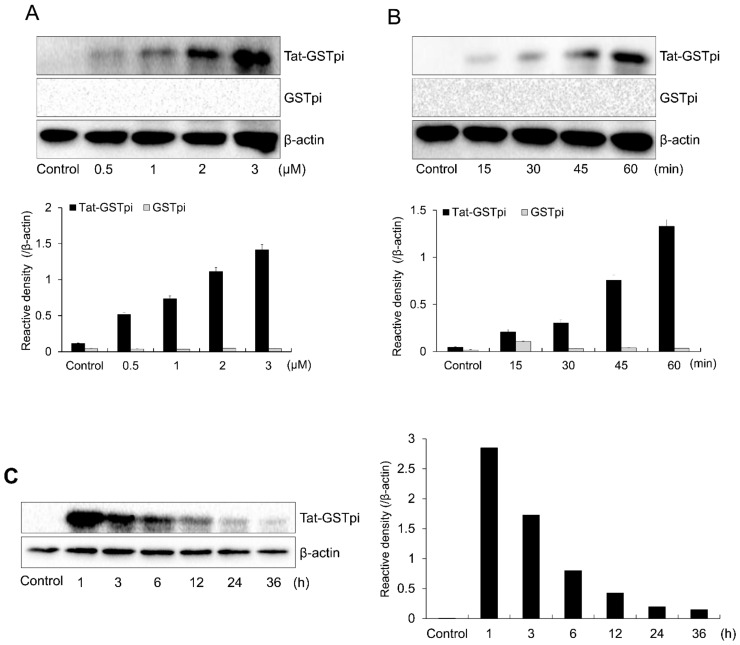
Delivery of Tat-GSTpi protein to SH-SY5Y cells. (**A**) SH-SY5Y cells culture media were treated with Tat-GSTpi or GSTpi protein at different doses (0.5–3 μM) for 1 h; (**B**) The cell culture media were treated with Tat-GSTpi (3 μM) or GSTpi protein for different time periods (15–60 min); (**C**) Intracellular stability of delivered Tat-GSTpi protein. The cell culture media were incubated for 36 h after delivery of Tat-GSTpi protein for 1 h. Then, Tat-GSTpi protein was detected by Western blotting and the intensity of the bands was measured by a densitometer. Data are represented as mean ± SEM (*n* = 3).

In order to deliver SH-SY5Y cells to the fusion proteins, we treated Tat-GSTpi proteins with various concentrations and incubation times (Figure 2A,B). Increasing the concentration and incubation time increased the amount of Tat-GSTpi protein detected using anti-His antibody. Furthermore, we showed that the delivered Tat-GSTpi protein was detectable for up to 24 h (Figure 2C). However, GSTpi protein was not delivered to the cells. These results indicate that the Tat-GSTpi proteins were delivered to the SH-SY5Y cells and were present in them for 24 h.

### 3.2. Delivered Tat-GSTpi Protein Inhibits MPP^+^-Induced SH-SY5Y Cell Damage

**Figure 3:**
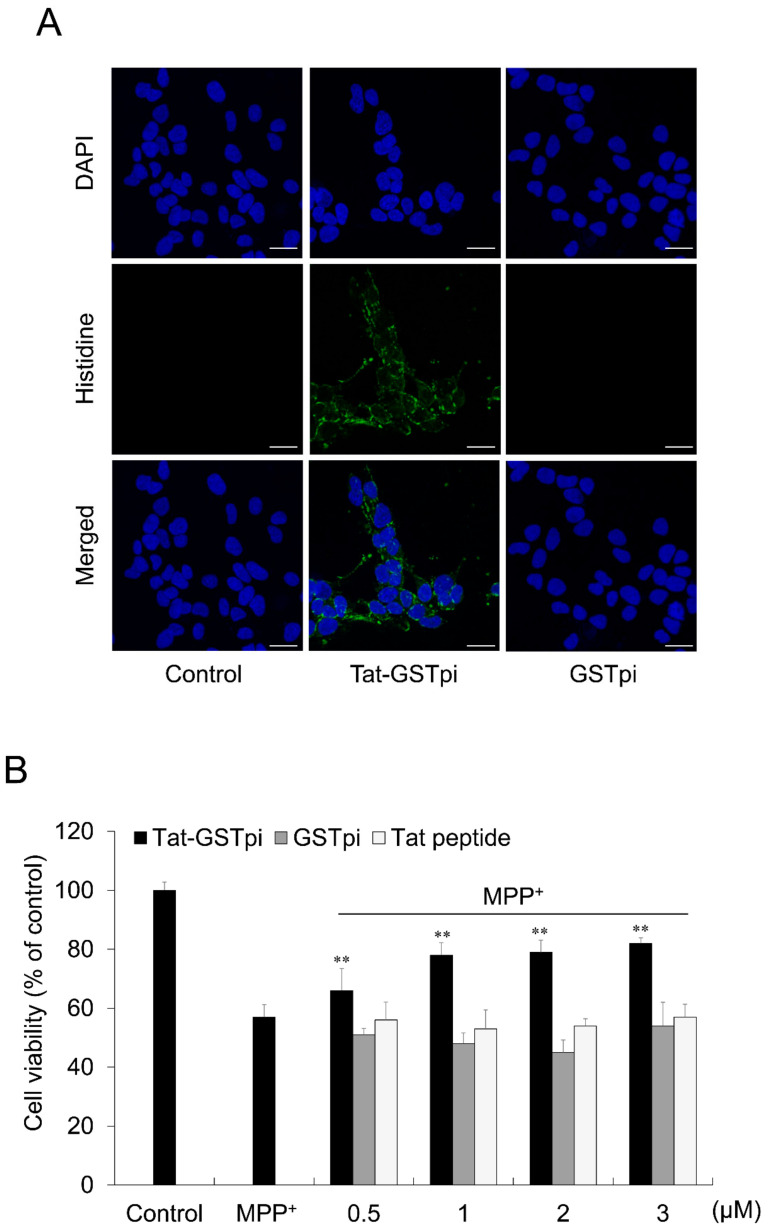
Effect of delivered Tat-GSTpi protein on MPP^+^-induced SH-SY5Y cell death. (**A**) The localization of delivered Tat-GSTpi protein was detected by confocal fluorescence microscopy. Scale bar = 20 μm; (**B**) Pretreatment of SH-SY5Y cells with Tat-GSTpi protein (3 μM), GSTpi protein, and Tat peptide for 1 h and treatment with 5 mM MPP^+^ for 14 h. Then, cell viabilities were estimated using MTT assay. Data are represented as mean ± SEM (*n* = 3). Scale bar = 50 μm. ** *p* < 0.01, compared with MPP^+^-treated cells.

To investigate the localization of Tat-GSTpi proteins in SH-SY5Y cells, immunofluorescence staining was performed (Figure 3A). Tat-GSTpi protein was localized in the cytoplasm and nuclei of SH-SY5Y cells. However, the GSTpi protein was not detected in the cells.

In order to validate the effect of the Tat-GSTpi protein on cell viability, an MTT assay was performed (Figure 3B). We showed that cell viability was not significant between the GSTpi protein and Tat peptide-treated cells compared with MPP^+^-only exposed cells, whereas the cell viability was significantly increased in proportion to the amount of protein in the Tat-GSTpi protein-treated cells.

**Figure 4:**
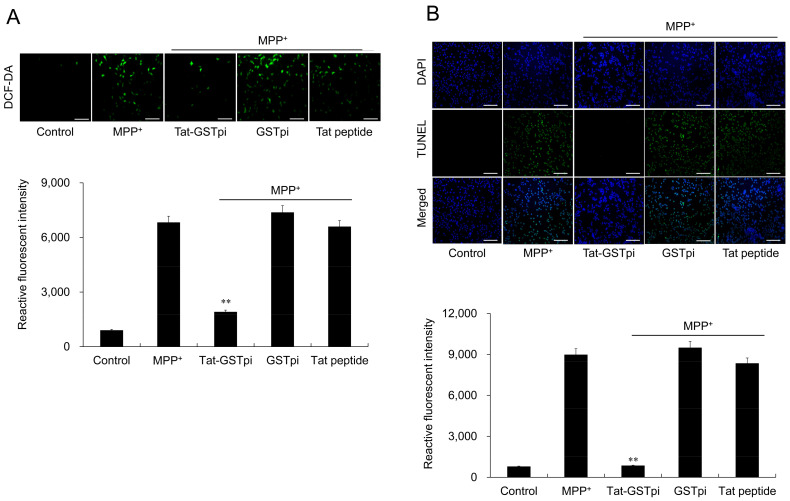
Effect of Tat-GSTpi protein on MPP^+^-induced cytotoxicity in the SH-SY5Y cells. (**A**) Cells were treated with Tat-GSTpi (3 μM), GSTpi protein, and Tat peptide for 1 h and exposed to MPP^+^ (5 mM) for 30 min. Intracellular ROS levels were determined by DCF-DA staining; (**B**) Cells were treated with Tat-GSTpi (3 μM), GSTpi protein, and Tat peptide for 1 h and exposed to MPP^+^ (5 mM) for 13 h. DNA fragmentation was detected by TUNEL staining. Fluorescence intensity was quantified using an ELISA plate reader. Data are represented as mean ± SEM (*n* = 3). Scale bar = 50 μm. ** *p* < 0.01, compared with MPP^+^-treated cells.

To assess the effect of Tat-GSTpi proteins on MPP^+^-induced ROS production and DNA damage in SH-SY5Y cells, DCF-DA and TUNEL staining was conducted (Figure 4A,B). The results showed that MPP^+^ markedly increased the amounts of ROS production and DNA damage and that the differences were not significant between the GSTpi protein and the Tat peptide-treated cells compared with MPP^+^-only exposed cells. However, the Tat-GSTpi protein reduced ROS production and DNA damage, indicating that the delivered Tat-GSTpi protein significantly inhibited cell death and reduced the levels of ROS and DNA damage.

### 3.3. Tat-GSTpi Protein Inhibits MPP^+^-Induced MAPK Activation in SH-SY5Y Cells

**Figure 5:**
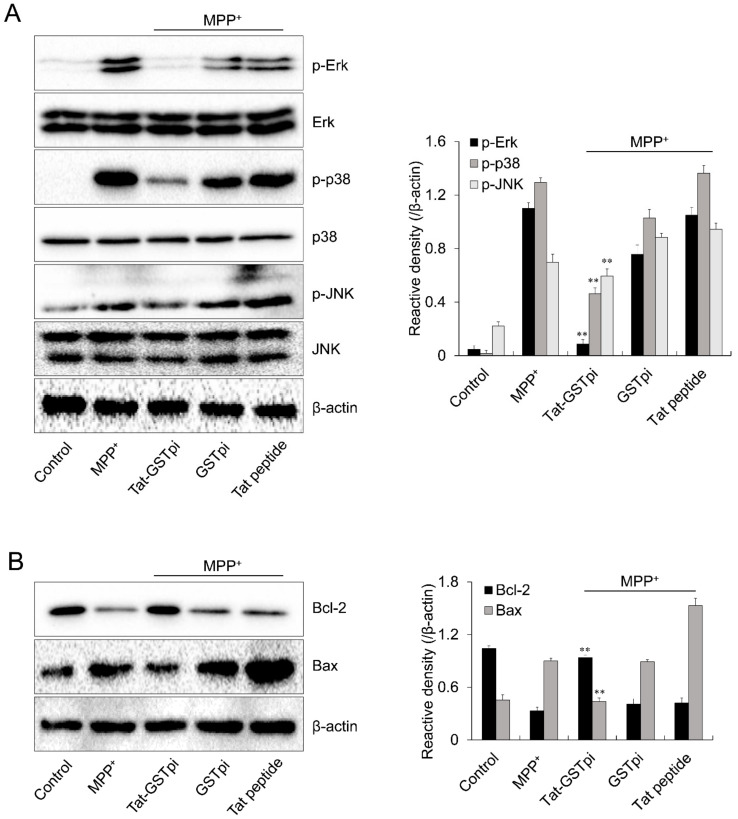
Effects of Tat-GSTpi protein on MPP^+^-induced expression of phosphorylation of MAPKs and apoptosis in SH-SY5Y cells. The cells were treated with Tat-GSTpi protein (3 μM), GSTpi protein, and Tat peptide for 1 h before being exposed to MPP^+^ (5 mM). The expression of phosphorylation of (**A**) MAPKs and (**B**) Bcl-2 and Bax levels were analyzed by Western blotting. Band intensity was measured by densitometer. Data are represented as mean ± SEM (*n* = 3). ** *p* < 0.01, compared with MPP^+^-treated cells.

Excessive ROS production induced the activation of the MAPK signaling pathway and led to cell death [38]. Thus, we investigated the effect of Tat-GSTpi proteins on the MPP^+^-induced MAPK signaling pathway in SH-SY5Y cells. The level of MAPKs phosphorylation was markedly increased in the MPP^+^-only exposed cells. Neither the GSTpi protein nor the Tat peptide affected MAPK signaling in the cells. However, the Tat-GSTpi protein inhibited the levels of MAPKs phosphorylation with MPP^+^ (Figure 5A).

We further investigated the effect of the Tat-GSTpi protein on Bax and Bcl-2 expression because MPP^+^-induced intracellular ROS can stimulate apoptotic signaling [39,40]. The expression level of Bcl-2 was reduced in MPP^+^-only exposed SH-SY5Y cells. However, the Tat-GSTpi protein increased the expression of Bcl-2. In contrast, the expression level of Bax was increased by MPP^+^ while the Tat-GSTpi protein reduced the expression level of Bax in MPP^+^-exposed cells (Figure 5B). Neither the GSTpi protein nor the Tat peptide affected the expression of Bcl-2 and Bax. Although further research on signaling pathways is needed, Tat-GSTpi proteins have a function in regulating MAPKs and apoptotic signaling through MPP^+^.

### 3.4. Tat-GSTpi Protein Protects Dopaminergic Neurons in the MPTP-Induced PD Model

**Figure 6:**
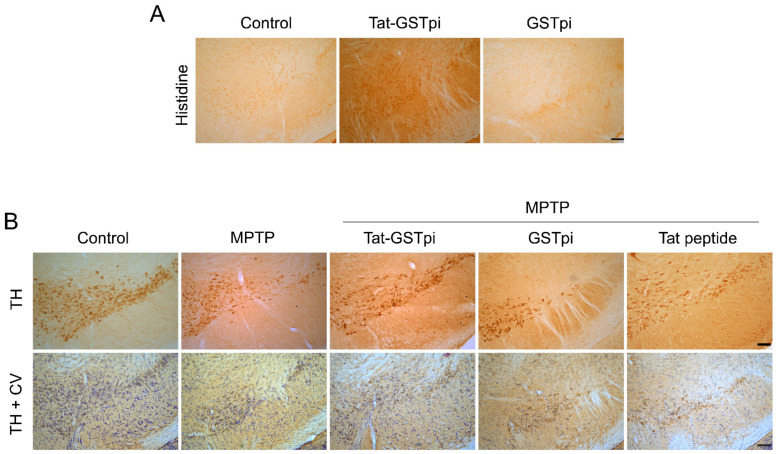
Effects of Tat-GSTpi proteins in an MPTP-induced PD animal model. (**A**) Delivery of Tat-GSTpi protein to the SN of mice brain. Tat-GSTpi protein or GSTpi protein was injected into mice (*n* = 7) at a dose of 2 mg/kg, and the brains were collected 12 h later. Brain tissues were immunostained with a rabbit anti-histidine antibody (1:400) and then stained with a biotinylated goat anti-rabbit secondary antibody (1:200). Scale bar = 100 μm; (**B**) Effect of Tat-GSTpi proteins against MPTP-induced dopaminergic neuronal cell death. Tat-GSTpi protein (2 mg/kg) was injected intraperitoneally into mice (*n* = 7), and the brains were collected 1 week after the injection of MTPT. Brain sections were immunostained with tyrosine hydroxylase (TH) immunoreactivity and double staining with cresyl violet (CV) and TH immunoreactivity. Scale bar = 100 μm.

In order to confirm the ability of Tat-GSTpi proteins to pass through the blood–brain barrier (BBB) of a mouse, Tat-GSTpi protein was injected intraperitoneally into mice and brain tissues were obtained 12 h after injection. Then, immunohistochemistry was performed (Figure 6A). In the Tat-GSTpi protein-treated group, strong immunoreactivity was present in the SN of the brain, while the control and GSTpi protein-treated groups were not immunoreactive. This result indicates that the Tat-GSTpi protein has the ability to cross the BBB of mice and get delivered to the brain.

In addition, to evaluate whether the delivery of Tat-GSTpi proteins to the brain could protect against dopaminergic neuronal cell death in the MPTP-induced PD model, we performed immunostaining with tyrosine hydroxylase (TH) antibody and cresyl violet (CV). In the Tat-GSTpi protein-treated group, strongly TH- and CV-immunoreactive cells were maintained in the MPTP-induced PD mice model. In contrast, GSTpi protein- and Tat peptide-treated groups were similar to the MPTP-treated group (Figure 6B). These results indicate that the Tat-GSTpi protein protected against dopaminergic neuronal cell death in the PD model.

## 4. Discussion

GSTpi proteins play an important role in cell survival due to its antioxidant functions [41]. However, the underlying mechanism and role of GSTpi proteins is not fully understood in PD. In this study, we showed that Tat-GSTpi protein delivered into cells can inhibit MPP^+^-induced cell death, ROS production, and DNA damage in SH-SY5Y cells by reducing oxidative stress and inhibiting MAPK phosphorylation. Furthermore, Tat-GSTpi proteins can be delivered to the brain tissue and protect dopaminergic neurons in the SN of the MPTP-induced PD mouse model.

In general, most proteins cannot be delivered into cells across the cell membrane. However, Tat PTD has been shown to have the ability to cross the cell membrane and be used to deliver protein into cells [42]. Although the precise mechanism of protein delivery is not yet clear, PTD fusion protein already has various therapeutic applications [24,25,26,27,28,29,30,31]. We revealed that Tat-GSTpi proteins can be delivered into SH-SY5Y cells and maintained in the cells for 24 h. Moreover, we showed that the PTD-GSTpi protein was ~HT22 cells in [32]. Although there were some differences in protein delivery to the cells, many reports have shown that therapeutic PTD fusion proteins can be delivered to cells [26,27,28,29,30,31]. The efficiency of the delivery of PTD fusion protein is known to depend on factors such as the type of PTD and cells. Furthermore, the delivery of therapeutic proteins into cells and tissues across the BBB is limited by the size and biochemical properties of the target proteins [24,42,43].

It is known that one of the etiologies of PD is that overwhelming elevate oxidative stress in the basal ganglia leads to dopaminergic neuron death. One study has observed elevated ROS levels and deficient antioxidant capacities in PD patients [44]. MPP^+^ induces increases in intracellular ROS in cells, which leads to cell death [37]. Thus, we validated the effect of Tat-GSTpi proteins on cellular toxicities and showed that MPP^+^ increased the amounts of ROS produced and the DNA damage in SH-SY5Y cells while the Tat-GSTpi protein reduced cellular toxicities and cell death in MPP^+^-exposed cells. Several studies have reported that MPP^+^ markedly increases intracellular dopaminergic ROS levels and cell death. Excessive production of ROS has also been shown to be involved in the pathological processes of neurodegenerative disorders, including PD [39,40,45,46]. Similarly, Smeyne et al. showed that MPP^+^-exposed in the SN primary dopaminergic neuronal cells caused cell death [47]. Some studies have also reported a decrease in GSTpi protein expression and a loss of dopaminergic neurons when transfected with GSTpi siRNA in dopaminergic neuronal cells [22]. On the other hand, other studies have reported that GSTpi protein levels are increased in SN of PD patients and suggested that GSTpi may be a biomarker for PD [21].

MAPK signaling pathways provide important functions in the mediation of MPP^+^-induced neurotoxicity in dopaminergic neurons and MPTP-induced PD mouse models. This evidence suggests that the regulation of MAPK activation is crucial in dopaminergic neuronal cell survival in PD [10,48,49,50]. Robust ROS production induced the activation of MAPK and apoptosis signaling pathways and led to cell death [46]. We found that the Tat-GSTpi protein inhibited the phosphorylation of MAPKs and apoptosis proteins in the MPP^+^ treatment cells, suggesting that the Tat-GSTpi protein has a function in regulating MAPKs and apoptotic signaling by MPP^+^. The JNK pathway, a member of the MAPK, is crucial for cell survival and inhibits apoptosis by various agents [51]. Wang et al. reported that phosphorylated JNK1 expression is a hallmark of cell death induction and that GSTpi proteins inhibit JNK-mediated phosphorylation of JNK in NIH3T3 cells and suggested that GSTpi proteins play a role in regulating kinase pathways [52]. We also found that PTD-GSTpi fusion proteins delivered to HT-22 cells inhibited expression levels of phosphorylated MAPKs and regulated apoptosis proteins in the HT-22 cells [32]. However, further studies are needed to provide insight into the mechanism by which GSTpi proteins inhibits dopaminergic neurons from oxidative stress.

Tat PTD has been widely studied and applied in protein delivery to various types of cells and brain tissues. Tat PTD fusion protein delivery to dopaminergic neuronal cells has been shown to cross the BBB and protect dopaminergic neurons in an MPTP-induced PD animal model [27,29,53,54]. The MPTP-induced PD mouse model has been used because of its similarity to human PD pathophysiology [45,55]. We showed that Tat-GSTpi proteins delivered to the brains of mice inhibited dopaminergic neuronal cell damage, indicating that the Tat-GSTpi protein had protective effects against cell death in PD. Consistent with our results, some studies have reported that the GSTpi protein inhibits the UV-induced apoptosis of SH-SY5Y neuroblastoma cells. In addition, GSTpi protein expression was shown to increase in the dopaminergic neurons of MPTP-treated mice and protect against MPTP-induced neuronal death, suggesting that the GSTpi protein may serve as an important protector of dopaminergic neurons [56,57].

## 5. Conclusions

In this study, we demonstrated that Tat-GSTpi proteins play a crucial role in protecting dopaminergic neuronal cells in MPP^+^- and MPTP-induced SH-SY5Y cells and in a PD animal model. These findings indicate that PTD-mediated delivery of GSTpi proteins can be an effective strategy for treating neuronal disease and further suggest that Tat-GSTpi proteins can be used to elucidate the roles and mechanisms within neuronal disease, including PD.

## Figures and Tables

**Table 1:** Information on the primary antibodies used for immunoblotting.

Name	Dilution	Source	Catalog Number
Anti-6X His tag^®^ antibody	1:5000	Abcam	ab9108
Phospho-p44/42 MAPK(Erk1/2)	1:2000	CST	#4376
p44/42 MAPK(Erk1/2)	1:2000	CST	#9102
Phospho-SAPK/JNK (Thr183/Tyr185)	1:1000	CST	#9251
JNK2 (56G8)	1:1000	CST	#9258
Phospho-p38 MAPK (Thr180/Tyr182)	1:2000	CST	#4631
p38 MAPK	1:2000	CST	#9212
Anti-Bcl-2	1:1000	Abcam	ab59348
Bax	1:1000	CST	#2772
β-Actin	1:10,000	CST	#4967

CST: Cell Signaling Technology.

## Data Availability

Data will be made available on request.
